# A Reduced Graphene Oxide Based Radio Frequency Glucose Sensing Device Using Multi-Dimensional Parameters

**DOI:** 10.3390/mi7080136

**Published:** 2016-08-05

**Authors:** Byeongho Park, Hyung Goo Park, Jae-hoon Ji, Jinsoo Cho, Seong Chan Jun

**Affiliations:** 1 School of Mechanical Engineering, Yonsei University, 50 Yonsei-ro, Seodaemun-gu, Seoul 120-749, Korea; enggis@naver.com (B.P.); zeustg@naver.com (H.G.P.); jupitersun@naver.com (J.J.); 2 Department of Computer Engineering, Gachon University, Gyeonggi-do 461-701, Korea; jscho@gachon.ac.kr

**Keywords:** glucose biosensor, multi-dimensional parameters, radio frequency, reduced graphene oxide

## Abstract

A reduced graphene oxide (RGO) based glucose sensor using a radio frequency (RF) signal is demonstrated. An RGO with outstanding electrical property was employed as the interconnector material between signal electrodes in an RF electric circuit, and it was functionalized with phenylbutyric acid (PBA) as a linker molecule to bind glucoses. By adding glucose solution, the fabricated sensor with RGO and PBA showed detecting characteristics in RF signal transmission and reflection. Frequency dependent electrical parameters such as resistance, inductance, shunt conductance and shunt capacitance were extracted from the RF results under the equivalent circuit model. These parameters also provided sensing characteristics of glucose with different concentrations. Using these multi-dimensional parameters, the RF sensor device detected glucose levels in the range of 1–4 mM, which ordinarily covers the testing range for diabetes or medical examination. The RGO based RF sensor, which fits well to a linear curve with fine stability, holds considerable promise for biomaterials detection, including glucose.

## 1. Introduction 

Glucose sensing from blood and urine is the usual diagnosis method for diabetes, and it is also used in the food industry, such as in fermentation for quality checking [1]. Various sensing techniques for glucose have been developed, such as fluorescence detection [2], electrochemistry [3], and surface-enhanced Raman scattering [4]. However, such techniques needed a mediator including enzymes or antibodies, which result in signal loss with electron- or photon-scattering [5,6]. To overcome this limitation, a single wall carbon nanotube with a high electrical property was employed as an interconnector in the sensor, which detected glucose with pico-molar sensitivity [7,8]. 

Similarly, reduced graphene oxide (RGO) with two-dimensional structure has also provided significant potential for biosensors, owing to its small signal loss under high electrical property and fine selectivity with specific binding affinity [9,10,11]. RGO has been applied to biosensors in several research areas, such as glucose sensors [12,13], gas sensors [14,15,16], and electrochemical bacteria sensors [17]. Aromatic ring structure and functional groups in RGO assist in connecting with the target material by π–π* stacking on its basal planes and chemical interaction [18]. Phenylboronic acid (PBA), which also has an aromatic ring structure, is a highly affinitive molecule with RGO, and it also makes the binding with glucose easily because of the diol group [19,20]. 

On the basis of the above considerations, we report here a development of the glucose sensor composed of RGO and PBA interconnector in a radio frequency (RF) system. In the RF measurement, scattering parameter (S-parameter), the ratio of output to input for RF signal transmission and reflection through the interconnector, was obtained in the range of 500 kHz to 4.5 GHz. Such measurement has exceptional characteristics for simple and rapid sensing by dropping small volume of solution sample and continuous real-time test with a wireless examination platform in the ultra-broadband microwave spectrum. In particular, the RF results in high frequencies provide remarkable and sensitive signal change with the resonance feature [21]. In addition, it can also be regarded as a valuable system since the decomposed multi–parameters, resistance (*R*), inductance (*L*), shunt conductance (*G*) and shunt capacitance (*C*) allow us to analyze the results more precisely [22]. 

## 2. Experimental Section

**Figure 1:**
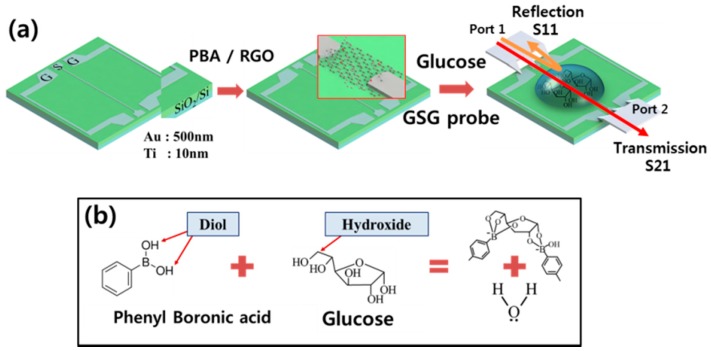
(**a**) Schematic of radio frequency (RF) measurement system with transmission and reflection between signal electrodes on ground-signal-ground (GSG) pattern (**b**) chemical reaction between phenylbutyric acid (PBA) and glucose.

D−(+) glucose (C_6_H_12_O_6_) was purchased from Sigma Aldrich Korea (Yongin, Korea), and we used several concentrations of glucose solution from 100 μM to 4 mM since the usual reference range of concentration in blood is close to 2.6 mM. The phosphate buffer tablets (10 mM phosphate, 150 mM sodium chloride, pH 7.3 to 7.5) were from Seoulin bioscience (Seoul, Korea). Our interconnector material composed of graphene oxide (GO) was prepared by the modified hummer method [23], and it was reduced by annealing, as in a previous work [24,25,26]. The high resistivity Si substrate purchased from Woori Material (Daejeon, Korea) with 500 nm dielectric layer (SiO_2_) for obtaining the stable electrical signal in RF was employed (Figure 1a). Gold electrodes (500 nm) were deposited by electron beam sputtering on the titanium adhesion layer (10 nm). Our electrode pattern had two signal (S) lines with a 4 μm gap on the center of the pattern and ground (G) lines, named ground-signal-ground (GSG). To connect the signal line using RGO as an interconnector, the Dielectrophoresis (DEP) method by alternating electric fields was used to focus the RGO flakes on the target position and to form the channel in a short time [15]. For the dielectrophoresis, RGO suspended solution (3 μL) was dropped on the middle of the substrate, and an alternating current (AC) through signal lines was applied under 10 V of potential and 10 KHz of frequency for 2 min.

For planting PBA on the RGO surface, 4-phenylbutyric acid (C_6_H_5_(CH_2_)_3_COOH, Sigma Aldrich, St. Louis, MO, USA) and 3-aminophenylboronic acid (C_6_H_8_BNO_2_, Sigma Aldrich, St. Louis, MO, USA) were dissolved in N,N-dimethylmethanamide (C_3_H_7_NO, DMF, Sigma Aldrich, St. Louis, MO, USA), and then N-(3-Dimethylaminopropyl)-N’-ethylcarbodiimide hydrochloride (C_8_H_17_N_3_·HCl, EDAC, Sigma Aldrich, St. Louis, MO, USA) was continuously added. We dropped this solution (10 μL) onto the RGO surface in the sensing device, and kept it for 2 h at room temperature. The functional groups of RGO assisted in attaching PBA on it with chemical interactions. After that, it was rinsed twice with pure DMF and distilled water to remove the reagents. Then, using a sensor device with PBA on RGO (PBA/RGO), we measured the S-parameter, which is the ratio of output to input signal between connected ports, by GSG probe with network analyzer (E5071C, Agilent, Santa Clara, CA, USA) after dropping glucose solution on the device surface. The network analyzer generated the microwave signal and detected the returning signal from the sensor device with GSG pattern. As shown in Figure 1a, the reflected S-parameter (S11) was obtained from port 1 to 1 again, and the transmitted S-parameter (S21) was from port 1 to 2. By the interaction between PBA and glucose molecule described in Figure 1b, glucose molecules were caught on the PBA/RGO interconnector.

For monitoring the structure of RGO on the substrate, we measured the field emission scanning electron microscope (SEM) (JSM-6701F, JEOL Ltd., Tokyo, Japan) and transmission electron microscope (TEM) (JEM-2010, JEOL Ltd., Tokyo, Japan) images, and atomic force microscope (AFM) (XE-BiO, Park Systems, Suwon, Korea). X-ray photoelectron spectroscopy (K-alpha, Thermo Fisher Scientific, Waltham, MA USA) was used to characterize the graphene based sample. 

## 3. Results and Discussion

**Figure 2:**
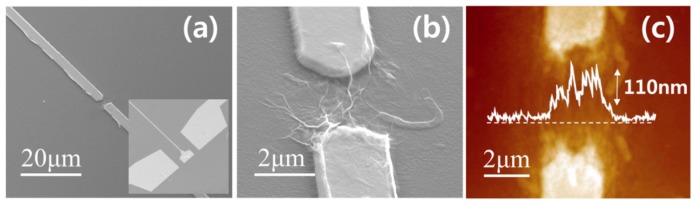
SEM image of (**a**) biosensor device with GSG pattern, (inset) signal line, and (**b**) PBA/reduced graphene oxide (RGO) interconnector part (**c**) AFM image of PBA/RGO interconnector.

Figure 2a,b present SEM images of stretched two signal lines and GSG electrode pattern. A PBA/RGO interconnector connected between two signal electrodes and PBA built up the entangled structure on the RGO sheet. The thickness of the PBA/RGO interconnector was 110 nm, as described in Figure 2c.

**Figure 3:**
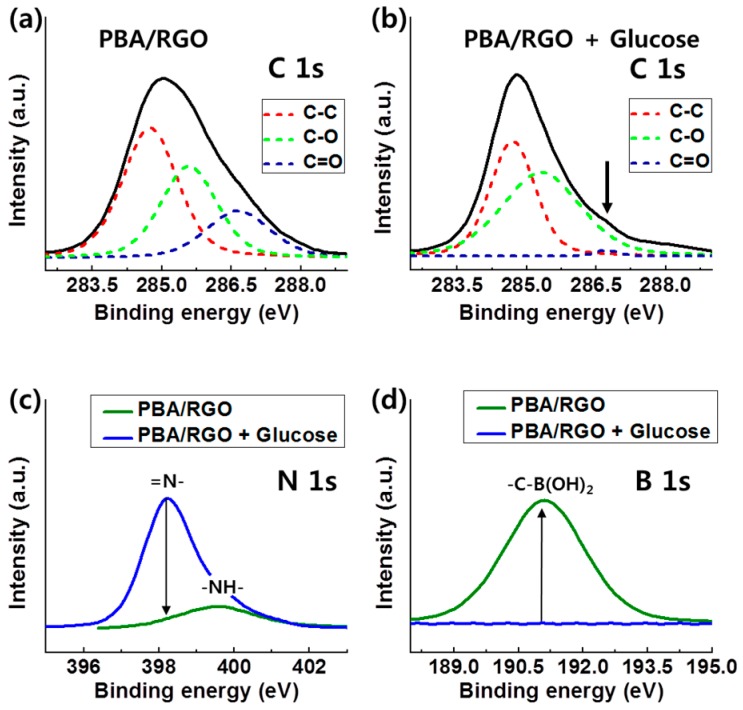
XPS results of PBA/RGO interconnector before and after adding glucose in (**a**,**b**) C 1s; (**c**) N 1s; and (**d**) B 1s orbital.

With C 1s, N 1s, and B 1s orbitals, Figure 3 represented the X-ray photoelectron spectra (XPS) results, which provides the information of chemical interaction, of PBA/RGO and glucose combined PBA/RGO. The C 1s results completely match with sp^2^ C–C bonding corresponding to 284.8 eV, C–OH/O–C–O, and COOH near 286 eV [27]; the intensity of sp^2^ at 284.8 eV was the most dominant in all features of the carbon based sample. PBA/RGO was reduced by adding glucose solution, and its shoulder peak at 286.7 eV decreased owing to new binding between PBA and glucose in the replacement of PBA/RGO functional groups [28,29]. The N 1s features consisted of three individual peaks appearing at 398.2 eV for =N–, at 399.7 eV for –NH–, and at 401.1 eV for NH_3_^+^. PBA/RGO exhibited mainly =N– feature at 398.2 eV from PBA before adding glucose. After glucose treatment, PBA/RGO showed decrement in =N– binding, and small –NH– features remain in the N 1s orbital of XPS characterization. In the B 1s, PBA/RGO with glucose presented –C–B(OH)_2_ feature at 191.6 eV [30]. In PBA/RGO, 3–aminophenylboronic acid that is able to interact chemically with glucose molecule has the nitrogen in amino and boron in boronic acid. The XPS features of these two atoms remarkably showed the existence of a functional group on PBA/RGO and interaction with glucose [31].

**Figure 4:**
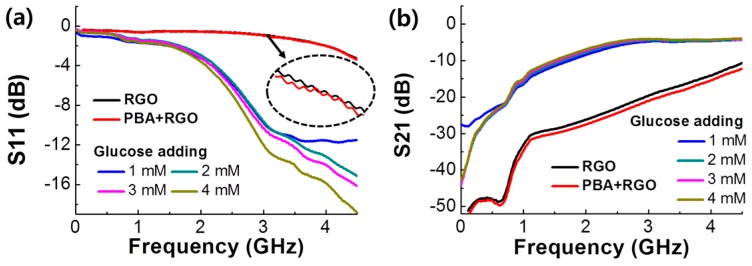
Up to 4.5 GHz, RF measurement result of RGO and PBA/RGO RF device. (**a**) Signal reflection (signal from port 1 to port 1, S11) and (**b**) transmission (signal from port 1 to port 2, S21). Glucose solution (3 μL) from 1 to 4 mM was dropped on the central position of the PBA/RGO device.

From two signal electrodes or ports on the RF device, a network analyzer can produce the input electrical power and receive the output power. The magnitude is determined by the ratio of output to input signal power. Each port, *i* and *j*, can provide four types of S-parameters such as S_11_, S_12_, S_21_, and S_22_. These parameters are the reflection (*i* = *j*) and the transmission (*i* ≠ *j*). S_11_ and S_21_ can be considered as a representative parameter individually to demonstrate all components in S parameters of the RF device. Figure 4 presents S-parameters of RGO and PBA/RGO devices with increasing glucose solution from 0 to 4 mM. According to the property of interconnector material, S-parameters were changed; intrinsic GO had similar characteristics to the open circuit, RGO and PBA/RGO showed RGO dominant S11 and S21 results (Figure S1). RGO based devices provided better signal transmission with higher conductivity.

After adding glucose solution, the transmission was significantly increased through the whole frequency range. The reflection parameter in Figure 4a showed that the reflection property did not considerably change under 1 GHz, and over 2 GHz the reflection magnitudes of glucose solutions were substantially decreased compared to that of the bare PBA/RGO line. PBA/RGO device provided considerable change in the S11 and S21 result after dropping glucose solution. Such S-parameter change indicates that the transmission increased with a decrement of signal loss in the interconnector. The interaction between PBA and glucose assisted in reducing the signal loss and the signal reflection in the PBA/RGO interconnector [32,33]. Many hydroxyl groups of the diol, which significantly reduce the conductivity of graphene and interfere signal transmission, in PBA/RGO were removed with condensation reaction of PBA and glucose [34]. As the concentration of glucose increases from 1 to 4 mM, S11 magnitude of PBA/RGO device was decreased. Although variations also occurred in the S21 result, it is too small to conceive as a sensor characteristic. 

## 4. Electrical Signal of RLGC

Since the S11 and S21 results provided too little variation to be used for sensor application, *R*, *L*, *G*, and *C* were extracted to investigate the specific sensor characteristics of the PBA/RGO RF device. From the S-parameter results, those four parameters were calculated with the following equation

(1) $$\left[\begin{array}{cc} A & B\\ C & D \end{array}\right]=\frac{1}{2S_{21}}\left[\begin{array}{cc} \left(1-S_{11}^{2}+S_{21}^{2}\right) & Z_{0}({\left(1+S_{11}^{2}\right)}^{2}-S_{21}^{2}\\ \frac{1}{Z_{0}}({\left(1-S_{11}\right)}^{2}-S_{21}^{2}) & \left(1-S_{11}^{2}+S_{21}^{2}\right) \end{array}\right]$$

where $Z_{0}$ indicate the initial load 50 Ω for GSG probing [35,36,37]. Then the impedance Z and RF propagation constant γ was computed by,

(2) $$\gamma =\cos^{-1}A,\;Z=\sqrt{\frac{B}{C}}$$

From telegrapher model, the properties of RF electronic circuit were obtained by de-embedding, removing the fixed effect and taking meaningful values from the measured results. The relationship between impedance, $Z=\sqrt{\left(R+j\omega L\right)/\left(G+j\omega C\right)}$ and propagation constant, $\gamma =\sqrt{\left(R+j\omega L\right)\left(G+j\omega C\right)}$ is composed of the standard electronic features of *R*, *L*, *G* and *C*. These features were extracted as follows,

(3) R=Re{γZ},L=Im{γZ}/ω,G=Re{γ/Z},C=Im{γ/Z}/ω

**Figure 5:**
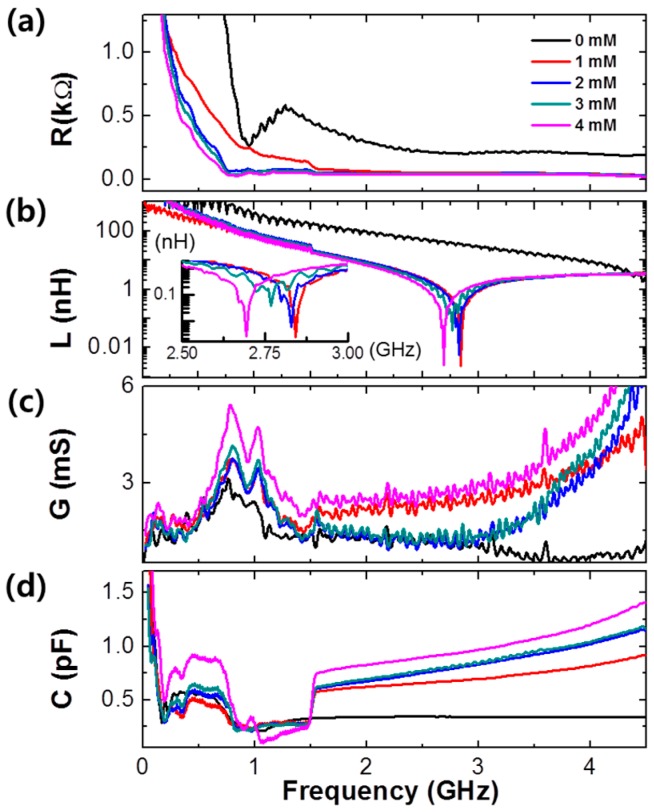
(**a**) Resistance, *R*; (**b**) inductance, *L*; (**c**) shunt conductance, *G*; and (**d**) shunt capacitance, *C*, of PBA/RGO device according to adding glucose with different concentrations from 0 to 4 mM.

*R* and *L* values were considerably related with the interconnector’s electrical properties, *G* and *C* values were obtained from the interaction between interconnector and substrate. These values are represented in Figure 5.

According to increasing frequency, the resistances were decreased since the electron transmission was significantly enhanced at high frequency, as shown in Figure 5a. Near the 2.75 GHz measurement frequency, the inductance result of PBA/RGO device showed a specific dip which originated from the resonance characteristic. 

As the concentration of glucose increased, the resistance (Figure 5a) generally decreased and the conductance (Figure 5c) and capacitance (Figure 5d) increased owing to the enhancement of conductivity with the RGO reduction, which resulted from the condensation reaction (Figure 1b) between PBA and glucose molecules. In particular, in Figure 5b, the inductance provided a resonance dip shifting according to glucose concentration. The dip shift to a lower frequency can be demonstrated through the relation between the resonance features and the viscosity of glucose solution [38,39]. Adding glucose on the PBA/RGO interconnector surface results in the enhancement of viscosity and acts as a damper, especially in the condition with the boronic acid [40]. Under the RF system, the conduction transferring the carrier to the opposite port was interfered with by the inductance and took a longer time when the inductance of the device had a high magnitude. 

**Figure 6:**
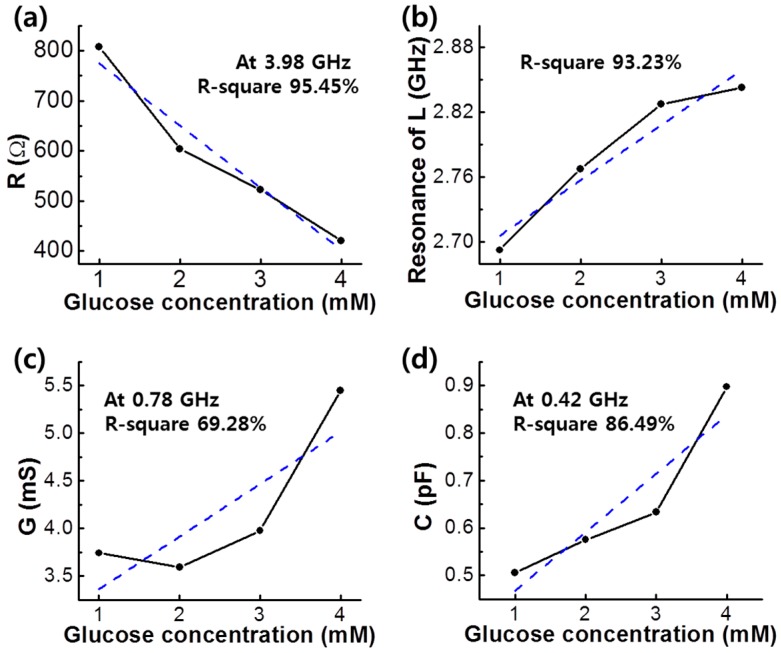
Linearity of (**a**) resistance, *R*; (**b**) resonance feature of inductance, *L*; (**c**) shunt conductance, *G*; and (**d**) shunt capacitance, *C*, of PBA/RGO device according to adding glucose with different concentrations from 1 to 4 mM.

Linearity of the PBA/RGO glucose biosensor was investigated with the R-square between the fitted results and experimental data according to glucose concentration. The linearity of the resistance result (Figure 6a) showed the highest linear response for glucose with 95.45% of R-square, and that of the inductance result (Figure 6b) corresponded to 93.23%. Shunt conductance and shunt capacitance, determined by the interaction between interconnector and substrate, provided worse linearity. 

Up to 100 μM level of glucose concentration, our PBA/RGO glucose sensor showed insufficient linearity with RF results, as shown in Table 1. Over the milli-molar concentration, the linear relationship between RF results and glucose concentration was remarkably revealed with high R-square value in the resistance and the resonance feature of inductance. Generally, linearity variations were increased under high glucose concentration. While the sensing ability of the resistance was balanced with a small range of error and fine linearity, the resonance feature of inductance exhibited unstable results with high fluctuation. The PBA/RGO glucose sensor showed a detection limit of 3 × 10^−5^ mol/L, which is higher than that of the electrochemical glucose sensor composed of a carbon nanotube [41]. 

## 5. Conclusions

In the present study, we demonstrated a glucose sensor using an RGO based RF system. RGO as an interconnector was functionalized with a PBA linker in order to catch the glucose molecules. The fabricated sensor showed detecting characteristics with fine linearity of the RF signal change as glucose solution was added. From measurement of the RF signal, several parameters such as *R*, *L*, *G* and *C* can be extracted by the equivalent circuit model. Under a high frequency domain, the RGO based RF sensor could sensitively detect small glucose concentration using multi-dimensional analysis with the four electrical parameters. The resistance was the most effective parameter for monitoring the glucose level with stable linearity and small fluctuation. The developed RF sensor can open up new possibilities in rapid and sensitive detection by applying various interconnector materials with specific binding affinity.

## Figures and Tables

**Table 1:** R-square of resistance (*R*), resonance feature of inductance (*L*), shunt conductance (*G*), and shunt capacitance (*C*).

Range of Glucose Concentration (mM)	Linearity (R-Square, %)			
	*R*	*L*	*G*	*C*
0.1–0.4	83.15 ± 4.22	69.04 ± 31.41	23.80 ± 2.28	21.51 ± 6.15
1–4	95.45 ± 9.60	93.23 ± 29.37	69.28 ± 42.54	86.49 ± 19.82

## Supplementary Materials

The following are available online at http://www.mdpi.com/2072-666X/7/8/136/s1, Figure S1: S-parameter results, (a) S11; (b) S21, of several electrodes with open without interconnector, GO, RGO, PBA/RGO.
