# Supplementary material for: A Reduced Graphene Oxide Based Radio Frequency Glucose Sensing Device Using Multi-Dimensional Parameters
Source: Micromachines (Basel). 2016 Aug 5;7(8):136. doi: 10.3390/mi7080136 (PMC6189738; doi:10.3390/mi7080136)
Supplement: Supplementary file 1 [file micromachines-07-00136-s001.pdf]

Supplementary Materials: Reduced Graphene Oxide Based Radio Frequency Glucose Sensing Device Using Multi-Dimensional Parameters

Byeongho Park, Hyung Goo Park, Jae-hoon Ji, Jinsoo Cho and Seong Chan Jun

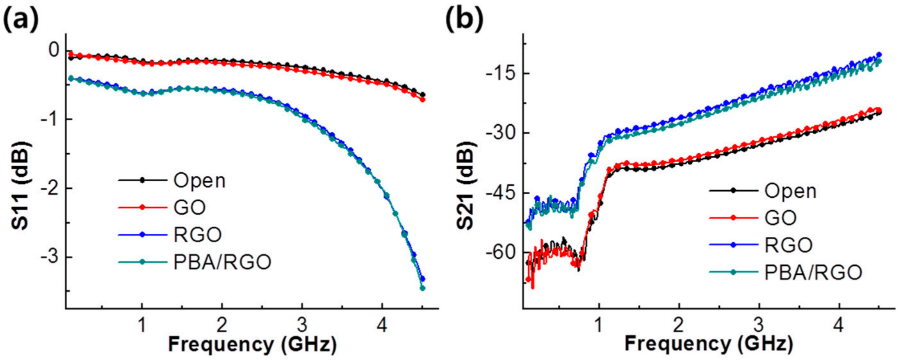

Figure S1. S-parameter results, (**a**) S11; (**b**) S21, of several electrodes with open without interconnector, GO, RGO, PBA/RGO.
